# Antimicrobial Photodynamic Therapy in the Approach of Complication After Implantation of Spiculated Polydioxanone Threads

**DOI:** 10.7759/cureus.42418

**Published:** 2023-07-25

**Authors:** Gabriela V Carrasco, Luciane Hiramatsu Azevedo, Alessandro C da Silva, Maristela M Lobo, Roger Kirschner, Patrícia Moreira de Freitas

**Affiliations:** 1 Restorative Dentistry, School of Dentistry/University of São Paulo, São Paulo, BRA; 2 Oral and Maxillofacial Surgery, Maxilart Private Clinic, São Paulo, BRA; 3 Especial Master’s Degree Course in Facial Esthetics, São Leopoldo Mandic, São Paulo, BRA; 4 Faculdade de Odontologia, Universidade de São Paulo, São Paulo, BRA

**Keywords:** facial cosmetics, complications, low power laser, antimicrobial photodynamic therapy, spiculated polydioxanone threads

## Abstract

The present manuscript is a clinical case report in which antimicrobial photodynamic therapy (aPDT) - using a low-power laser (660 nm) associated with methylene blue photosensitizer (0.01%) - was considered for treating a case of complication after implantation of spiculated polydioxanone (PDO) threads, an aesthetic procedure worldwide performed in facial cosmetic non-surgical procedures. A 72-year-old female patient presented an infection in the face region where the PDO thread was implanted (mandible angle). After divulsion and local drainage, it was decided to associate aPDT using a low-level laser associated with a 0.01% methylene blue photosensitizer. Two sessions of aPDT were performed - on sequential days - and within 48 hours the region was dry and without signs of secretion. The use of aPDT seems to be a promising and effective option in cases of infections after implantation of PDO threads, consisting of a safe technique, of low execution complexity and without adverse effects.

## Introduction

During the last decades, non-surgical aesthetic procedures have been growing exponentially. According to a survey carried out by the Brazilian Society of Plastic Surgery (BSPS), they rose from 17.4% (2014) to 49.9% (2018) [1]. Since 2019, as Orofacial Cosmetic (non-surgical cosmetic procedures) was introduced as a certification course of Dentistry in Brazil, many of these procedures became part of the dental routine.

According to the same survey carried out by BSPS [1], in 2018, the use of spiculated polydioxanone threads (sPDO) became the fourth most performed non-surgical procedure in Brazil, after botulinum toxin application, dermal fillers, and peelings [1].

The use of sPDO threads aims to lift the ptosed facial tissues in a non-surgical fashion. This procedure can be performed in an outpatient setting and is a non-surgical and non-invasive procedure. It can be done under local anesthesia and has an immediate result (lifting effect) [2]. In addition, it has a short post-procedure recovery period when compared to other surgical procedures with the same purpose such as a regular surgical facelift [2]. However, it has limited long-lasting results (average of 6-12 months) and a high incidence of minor complications (average of 30%) [3].

There are several types of facial suspension threads on the market, but the most commonly used are threads made of polydioxanone (PDO), which are considered to be safe and effective for aesthetic purposes [3]. PDO threads have also other advantages such as compatibility with human biological tissues, non-allergenic nor pyogenic [2], and they are also absorbable by the body [4]. Due to its metabolic degradation and absorption processes, threads can stimulate the layers of skin. Fibroblast activation initiates new collagen types I and III formation, improving skin density and quality in the long term [3].

Despite being considered a safe and relatively simple procedure, there is a risk of minor to moderate intraoperative and postoperative complications related to the implantation of sPDO threads. Pain, tingling, erythema, hematoma, and edema are the most common immediate postoperative complications, being natural and expected reactions from the body [2]. In addition, complications such as infection, translucency, thread migration, scar formation, and facial asymmetry may also occur with minor incidents. Among the minor complications related to sPDO procedures, infection with the possibility of tissue necrosis and hypo/hypertrophic scar formation is one of the most undesired and, in some cases, with limited resolution.

An alternative approach to control local infection with rapid resolution is the use of antimicrobial photodynamic therapy (aPDT) using low-power lasers combined with photosensitizing agents, with effectiveness in microbial reduction. When laser light (660 nm) interacts with the photosensitizer (e.g., methylene blue), reactive oxygen species are formed and degrade the microbial cell membrane and its DNA [5].

This case report aims to show and discuss, to the best of our knowledge, the first report in the literature of a clinical case in which aPDT was the option for a minimally invasive approach with no adverse effects of infection in the region of insertion of sPDO threads.

## Case presentation

Patient C.S.R., female, 72 years old, was referred to the laser clinic for the treatment of a complication after the implantation of an sPDO thread. The patient did not report any comorbidity regarding medical records; she only reported an allergic reaction to iodine. Likewise, the patient did not present any dental condition - assessed by anamnesis and intra-oral examination - of relevance to the clinical condition presented on the face.

In November 2021, the patient underwent a facelift procedure with lifting threads (PDO, spiculated, I-Thread, Korea). Two threads were inserted in mandible angle areas (bilateral) and the knot was made between these two threads. The procedure was uneventful, with no intraoperative complications reported.

One month after the sPDO thread implantation (December/2021), the patient began to feel discomfort at the right side of the mandibular angle (where the incision was made for the thread implantation). In addition to discomfort, the patient reported a firm and palpable nodule in the same region. Because at that time the patient did not have any signs or symptoms of infection, she was treated with injections of hyaluronic acid, to accelerate the degradation processes of the resorbable material (sPDO). Thirty days after this initial treatment (January/2022), the patient continued to report discomfort and tenderness in the same area and then, she was submitted to plasma jet treatment (Plasmed, Ibramed, Amparo, SP, Brazil) to stimulate tissue remodeling and repairing.

Three months after the sPDO treatment (February/2022), the patient was referred to the clinic of the Special Laboratory of Lasers in Dentistry of the University of São Paulo (LELO-FOUSP). At this time a small area of infection was detected in the region of the right mandibular angle, with an abscess, restricted to the orifice region of implantation of sPDO threads.

**Figure 1:**
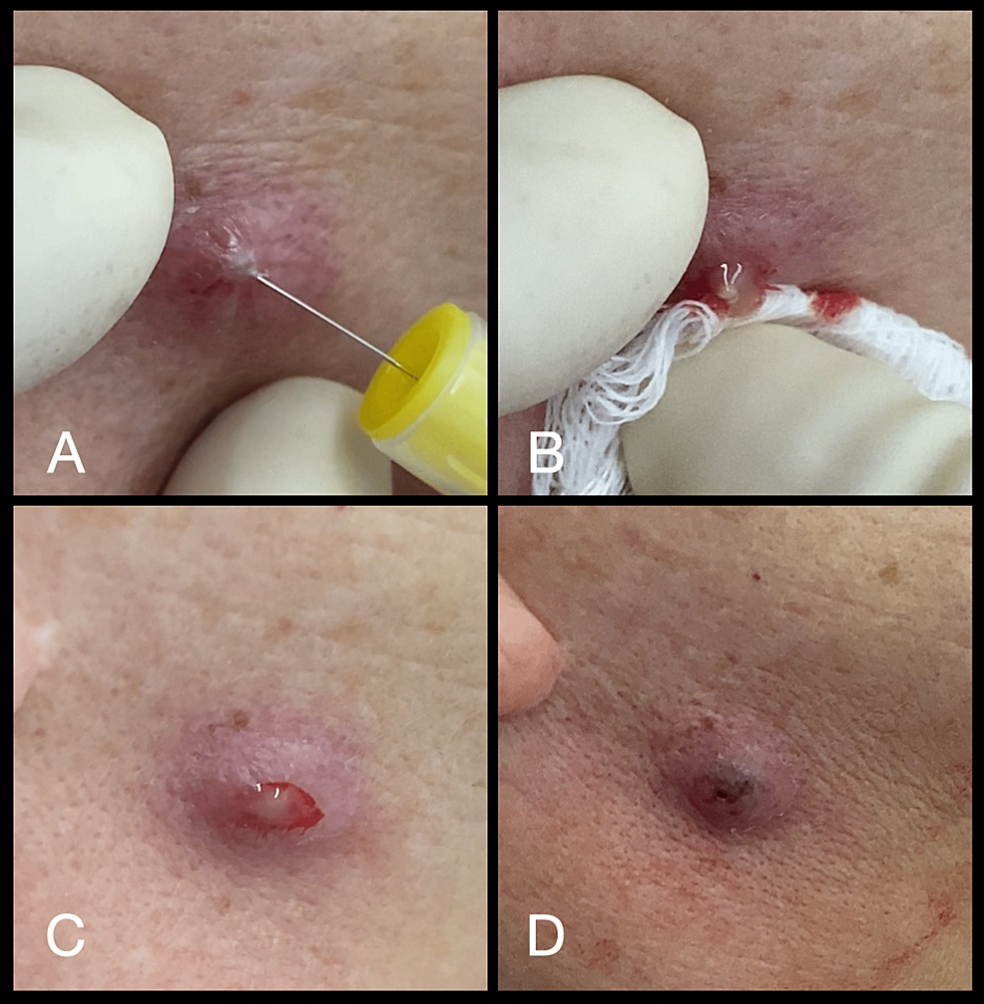
(A) Tissue divulsion with a sterile needle; (B) Manual area compression and drainage; (C) Purulent secretion present in the orifice region of implantation of sPDO threads; (D) Drainage completed. sPDO: spiculated polydioxanone

**Figure 2:**
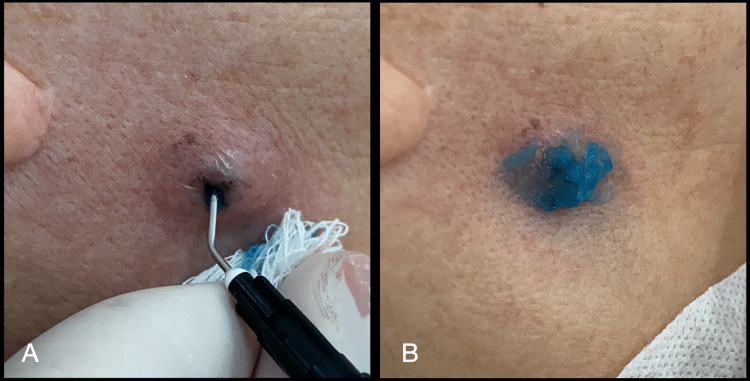
(A) Application of 0.01% methylene blue solution in the skin; (B) Application of 0.01% methylene blue gel on the skin surface.

**Figure 3:**
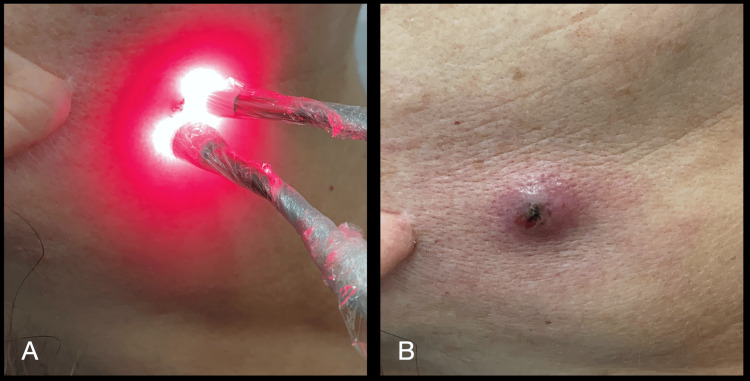
(A) After waiting for the pre-irradiation time (5 min), laser irradiation was performed with emission in the red spectrum (660 nm), with 6 J per point, in 6 points. (B) Immediately after the first aPDT session. aPDT: antimicrobial photodynamic therapy

At the first clinical session, the patient was not taking any medication. Divulsion was performed with a sterile needle (Figure 1A), and the purulent secretion was drained (Figures 1B-1D). After, aPDT was performed (Figures 2-3), following the protocol described in Table 1.

**Table 1:** Information on the antimicrobial Photodynamic Therapy (aPDT)

Products	Features	Manufacturer	Protocol
Photosensitizer (gel)	0.01% methylene blue	Biolux, CLSP, São Paulo, SP, Brazil	Application of 0.01% methylene blue solution inside the skin; Application of 0.01% methylene blue gel on the skin surface; 5 minutes of pre-irradiation time (no use of light); Low power laser irradiation (660 nm), power 100 mW, energy 6 J per point, 6 points (0.5 cm between points of irradiation), 60 seconds per point of irradiation, energy density of 214.3 J/cm^2^. Removal of gel excess from the surface of the skin with sterile gauze. After 24 hours, a second session of aPDT was performed, following the same protocol described above.
Photosensitizer (solution)	0.01% methylene blue	Biolux, CLSP, São Paulo, SP, Brazil	
Low Power Laser (Diode laser, AlGaAs)	Emission in the red spectrum (660 nm), 0.028 cm^2^	Therapy EC (DMC, São Carlos, SP, Brazil)	

**Figure 4:**
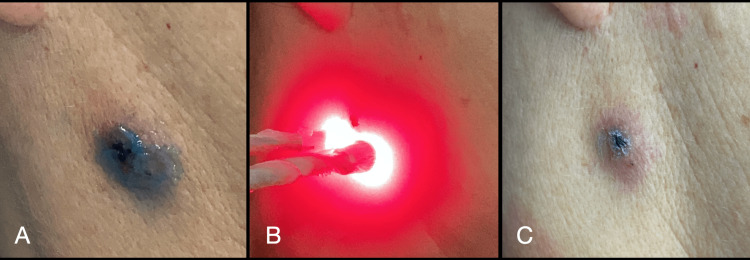
(A) Application of 0.01% methylene blue solution and gel inside and on the skin, respectively; (B) After waiting for the pre-irradiation time (5 min), laser irradiation was performed with emission in the red spectrum (660 nm), with 6 J per point, in 6 points. (B) Immediately after the second aPDT session. aPDT: antimicrobial photodynamic therapy

After 24 hours, the patient returned for reassessment and the region was dry, less swollen, and with no signs of secretion. Even so, it was decided to do a second session of aPDT, to minimize the risks of the evolution of a new infectious condition, as shown in Figure 4.

**Figure 5:**
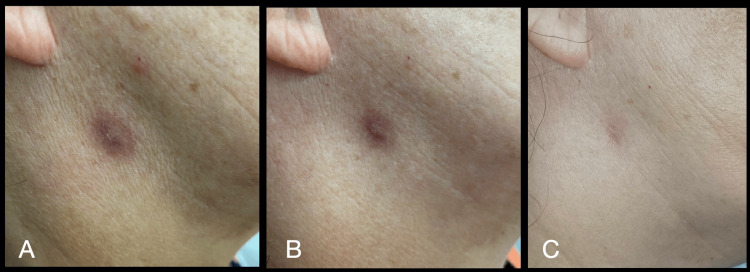
Area of the lesion: (A) Ten days after the second aPDT session; Lesion area: (B) Sixteen days after the second aPDT session; (C) Seven months after the second aPDT session. aPDT: antimicrobial photodynamic therapy

The evaluations were carried out in the following weeks, with a very satisfactory clinical evolution of the initial infection (Figures 5A-5C).

## Discussion

Facial rejuvenation with PDO threads is a safe and effective procedure, not commonly associated with major complications [5]; however, like any procedure, there are risks. One of the main undesired risks in non-surgical facial cosmetic procedures (Orofacial Harmonization) is necrosis, resulting from infections. Studies show that infections account for approximately 0.7% [6] to 8.9% [7] of the complications of such procedures. Authors who reported 0.7% of complications of this type [6] chose to prescribe post-procedure broad-spectrum antibiotic therapy. Although this type of complication is rare, it is the major cause of thread removal [3], in addition to being more common in patients over 50 years of age [3], as in this case.

These infections can be caused both by the negligence of the professional during the procedure (breaking of the septic chain) and also by the patient who may not follow the recommendations of the responsible professional [3].

Complications demand a quick diagnosis and immediate treatment to reduce patient discomfort and reduce the risk of morbidities or unsightly sequelae [2]. Within this context, whenever possible, early and minimally invasive procedures should be considered. Therefore, for a case of local infection, as described in the present clinical report, the aPDT protocol can be proposed, representing a non-invasive, relatively low-cost, and low-complexity approach.

The photosensitization mechanism of aPDT consists of the interaction of light with the photosensitizer and oxygen, generating free radicals that induce severe damage to microbial cells, leading to their microorganism’s apoptosis. Light is responsible for exciting the photosensitizing agent, which interacts with neighboring molecules through two mechanisms [8]. The photosensitizer in the excited state can act by removing a hydrogen atom from a molecule of the biological substrate (phospholipids, cholesterol, proteins, among others) [8] or by transferring electrons, generating radical ions that tend to react with oxygen in the ground state. Oxidized products responsible for the free radical chain are formed, such as superoxide radical (O2-), hydrogen peroxide (H2O2), and hydroxyl radical (OH), which can oxidize a wide variety of biomolecules. The excited state photosensitizer can also transfer energy to ground-state molecular oxygen, producing singlet oxygen. This is the dominant mechanism in aPDT [9]. Singlet oxygen is a highly reactive form of oxygen and is considered the main mediator of the photochemical damage caused to microorganisms by many photosensitizers [10].

For aPDT to occur, photons must interact with the photosensitizer [10]. There are several effective photosensitizers for aPDT. The best known for dentistry is methylene blue (MB), whose maximum absorption occurs at 664 nm, that means, PDT with MB must use light sources emitting photons in the visible red range, such as low-power lasers with emission in the spectrum of 660 nm [10].

In skin pathologies, the use of aPDT is already widespread and of remarkable effectiveness [11]. Multicenter randomized controlled studies [11] demonstrate the high efficacy and superior final cosmetic result of this therapeutic modality in relation to conventional treatments with systemic medication. For non-oncological skin conditions, such as acne vulgaris, viral warts, and localized scleroderma, there are also reports and case series confirming the therapeutic potential of photodynamic therapy [11]. Likewise, other authors [12] describe that PDT is an effective protocol for the treatment of superficial mycoses [12].

To date, there is no report in the literature on the application of photodynamic therapy for complications resulting from a facelift with sPDO threads. Clinical studies should be carried out to assess the benefits and effectiveness of this therapy in complications following non-surgical cosmetic procedures (Orofacial Harmonization), expanding the options for a minimally invasive approach for professionals working in this specialty.

## Conclusions

This study, to the best of our knowledge, presented the first report in the literature of a clinical case in which aPDT was the option for a minimally invasive approach to infection in the face region where sPDO threads were implanted. The therapy has been worldwide indicated for viral, fungal, and bacterial infections and is a result of the laser light (660 nm) interaction with the photosensitizer (e.g., methylene blue), forming reactive oxygen species capable of degrading the microbial cell membrane and its DNA. In the present case, only two sessions of aPDT - performed with an interval of 24h - has been shown as a promising and effective option in cases of complications from infections after implantation of sPDO threads, consisting of a safe and feasible technique, low cost and without side effects.

## References

[1] (2022). 2018 Census. Sociedade Brasileira de Cirurgia Plastica (Brazilian Society of Plastic Surgery). Sociedade Brasileira de Cirurgia Plastica (Brazilian.

[2] Tavares JP, Oliveira CA, Torres RP, Bahmad F Jr (2017). Facial thread lifting with suture suspension. Braz J Otorhinolaryngol.

[3] Niu Z, Zhang K, Yao W (2021). A meta-analysis and systematic review of the incidences of complications following facial thread-lifting. Aesthetic Plast Surg.

[4] Suh DH, Jang HW, Lee SJ, Lee WS, Ryu HJ (2015). Outcomes of polydioxanone knotless thread lifting for facial rejuvenation. Dermatol Surg.

[5] Foote CS (1991). Definition of type I and type II photosensitized oxidation. Photochem Photobiol.

[6] Sarigul Guduk S, Karaca N (2018). Safety and complications of absorbable threads made of poly-L-lactic acid and poly lactide/glycolide: experience with 148 consecutive patients. J Cosmet Dermatol.

[7] Li YL, Li ZH, Chen XY, Xing WS, Hu JT (2021). Facial thread lifting complications in China: analysis and treatment. Plast Reconstr Surg Glob Open.

[8] Correia JH, Rodrigues JA, Pimenta S, Dong T, Yang Z (2021). Photodynamic therapy review: principles, photosensitizers, applications, and future directions. Pharmaceutics.

[9] Fitzgerald F (2017). Photodynamic Therapy (PDT): Principles, Mechanisms and Applications. https://novapublishers.com/shop/photodynamic-therapy-pdt-principles-mechanisms-and-applications/.

[10] Wan MT, Lin JY (2014). Current evidence and applications of photodynamic therapy in dermatology. Clin Cosmet Investig Dermatol.

[11] Shen JJ, Jemec GB, Arendrup MC, Saunte DM (2020). Photodynamic therapy treatment of superficial fungal infections: a systematic review. Photodiagnosis Photodyn Ther.

[12] Philipp-Dormston WG (2018). Photodynamic therapy for aesthetic-cosmetic indications. G Ital Dermatol Venereol.

